# Text-guided few-shot liver and tumor segmentation

**DOI:** 10.3389/fdgth.2026.1823870

**Published:** 2026-05-29

**Authors:** Hongling Chen, Aibing Xu, Li Zhang, Miao Ouyang, Zhonghang Zhu, Jiacheng Wang

**Affiliations:** 1 Nantong Tumor Hospital/Nantong University Affiliated Tumor Hospital, Nantong, Jiangsu, China; 2 School of Electronic Information, Wuhan University of Science and Technology, Wuhan, China; 3 Manteia Technology, Co., Ltd., Xiamen, Fujian, China

**Keywords:** automated semantic guidance, cross-dataset generalization, digital oncology, few-shot learning, liver tumor segmentation, vision-language models

## Abstract

**Introduction:**

High-precision liver and tumor segmentation is a cornerstone of digital oncology, yet its clinical deployment remains constrained by two persistent challenges: the scarcity of pixel-level annotations and severe performance degradation under cross-center domain shift. Although few shot learning offers a promising direction for data-efficient modeling, existing approaches relying solely on visual similarity often fail to generalize across heterogeneous clinical environments.

**Method:**

In this study, we propose a text-guided few-shot segmentation framework that integrates clinical semantic information as an explicit inductive bias to bridge the gap between low-level pixels and high-level diagnostic reasoning. The framework consists of three synergistic modules: an Automated Semantic Generator that employs a large-scale vision-language model to encode clinically structured semantic descriptions into structured linguistic priors. A Text-Guided Gating (TGG) mechanism that adaptively modulates visual representations to filter scanner-dependent artifacts, and a Decoupled Prototype Learner that constructs unbiased class prototypes via per-image averaging and gradient detachment to address extreme class imbalance. Together, these components transform segmentation from a pure visual matching task into a knowledge guided reasoning process, ensuring both data efficiency and improved cross-dataset robustness across heterogeneous datasets.

**Results:**

Experiments on LiTS and 3DIRCADb show that the method consistently outperforms state-of-the-art supervised, foundation-model-based, and few-shot baselines. On 3DIRCADb, compared to PANet, the strongest few-shot baseline, our method improves external liver Dice by 8.7 percentage points and external tumor Dice by 26.3 percentage points, effectively mitigating performance degradation in conventional supervised models.

**Discussion:**

These results demonstrate that cross-modal semantic guidance enhances robust medical image segmentation under domain shift.

## 1 Introduction

Accurate segmentation of the liver and hepatic tumors from Computed Tomography (CT) images is a cornerstone task in digital oncology, underpinning a wide range of downstream clinical applications, including surgical planning, radiotherapy dose optimization, and longitudinal monitoring of treatment response [1–3]. Given the global burden of primary liver cancer and metastatic liver disease, robust and generalizable automated segmentation tools are of critical importance for scalable and reproducible clinical decision support systems. Over the past decade, deep learning–based segmentation methods have achieved remarkable progress, with encoder–decoder architectures derived from U-Net [4] becoming the dominant paradigm in medical image segmentation [5, 6]. More recently, transformer-based and foundation model–driven approaches, such as SAM-inspired architectures, have further advanced segmentation accuracy under fully supervised settings [7]. However, despite their strong performance on benchmark datasets, these models often exhibit substantial performance degradation when deployed in real-world clinical environments.

Such degradation can largely be attributed to two fundamental challenges. First, high-quality voxel-wise annotation of liver tumors is labor-intensive and requires experienced radiologists, leading to limited availability of labeled data and restricting scalability across institutions. Second, heterogeneity in imaging protocols, scanner manufacturers, reconstruction parameters, and patient demographics introduces pronounced distribution shifts across datasets. Models trained on data from a single center frequently fail to generalize to unseen domains, a phenomenon extensively documented in recent studies on domain shift and domain generalization in medical imaging [8–10]. Together, annotation scarcity and domain shift pose significant barriers to the clinical translation of segmentation models.

To mitigate these challenges, prior research has explored a range of strategies, including transfer learning from large-scale datasets [11–13], domain adaptation through adversarial [14, 15] or feature alignment techniques [16, 17], and domain generalization via data augmentation [18, 19] or invariant representation learning [20, 21]. While effective in controlled scenarios, these approaches often depend on access to auxiliary datasets, target-domain samples, or complex training pipelines, limiting their practicality in clinical workflows that demand minimal annotation overhead and rapid deployment.

In this context, Few-Shot Learning (FSL) has emerged as a promising paradigm for medical image segmentation, aiming to adapt models using only a handful of annotated samples from a target domain. Existing few-shot segmentation methods predominantly rely on metric-based matching [22, 23] or prototype learning [24], where segmentation is guided by visual similarity between support and query images [25, 26]. However, such approaches primarily operate at the level of pixel intensities or low-level texture features, which are highly sensitive to imaging noise and acquisition variability. As a result, their robustness across datasets and institutions remains limited.

In contrast, clinical decision-making by radiologists extends beyond local visual patterns. Lesion identification and delineation are informed by higher-level semantic knowledge, including morphological characteristics, relative spatial relationships, density patterns, and contextual cues from surrounding anatomical structures. These semantic attributes tend to be more stable across imaging conditions and play a critical role in reducing inter-observer variability. Despite their importance, such clinically meaningful semantic information has not been systematically incorporated into existing few-shot segmentation frameworks.

Recent advances in Vision–Language Models (VLMs) offer a new opportunity to bridge this gap. By jointly modeling visual content and natural language, VLMs enable the extraction of structured, high-level semantic descriptions from images, providing a complementary representation beyond raw visual features [27, 28]. In this work, we leverage a state-of-the-art VLM, Qwen2.5-VL [29], to automatically generate fine-grained textual descriptions of CT images without requiring additional manual annotation. These descriptions encode clinically relevant semantic cues, such as lesion appearance, boundary characteristics, and spatial relationships, and can be interpreted as concept-level priors that are inherently more robust to domain variations.

Building on this insight, we propose a text-guided few-shot liver tumor segmentation framework that integrates language-derived semantic priors with visual representations through a Text-Guided Gate (TGG) and a decoupled prototype learning strategy. This design enables effective fusion of complementary modalities while preserving the flexibility required for few-shot adaptation. We evaluate the proposed method on the LiTS and 3DIRCADb benchmarks and compare it against a diverse set of representative segmentation approaches, including nnU-Net [30], UNet [4], MedNeXt [31], SegResNet [32], and other models. Extensive experiments demonstrate consistent performance gains, particularly under cross-dataset and external validation settings, highlighting the enhanced generalization capability of the proposed framework.

Overall, this study demonstrates that automated semantic guidance derived from vision–language models can serve as an effective inductive bias for few-shot medical image segmentation. By moving beyond purely visual matching paradigms and aligning more closely with clinical reasoning processes, the proposed approach offers a practical and scalable pathway toward data-efficient and generalizable segmentation models in digital health.

## 2 Related work

### 2.1 Evolution of medical image segmentation

Medical image segmentation has undergone a paradigm shift driven by the success of deep convolutional neural networks (CNNs), with the U-Net architecture establishing a foundational framework for a wide range of clinical applications [4]. Subsequent extensions, such as Attention U-Net [33], introduced spatial and channel-wise gating mechanisms to enhance feature discrimination by suppressing irrelevant background regions. Meanwhile, nnU-Net [30] demonstrated that careful automation of the entire training pipeline—including preprocessing, architecture configuration, and post-processing—can yield strong and reproducible performance across diverse segmentation tasks.

More recently, the incorporation of Transformer-based architectures has enabled more effective modeling of long-range dependencies and global context. Representative methods such as TransUNet [34] and SwinUNETR [35] integrate self-attention mechanisms into encoder–decoder designs, leading to improved delineation of large anatomical structures and complex organ boundaries. Despite these advances, the majority of existing segmentation models remain fully supervised and heavily reliant on large-scale, pixel-wise annotations. Moreover, their performance often degrades substantially when applied to data acquired under different imaging protocols or from unseen clinical centers, highlighting persistent challenges related to annotation scarcity and domain shift [8, 36].

### 2.2 Few-shot learning in the medical domain

To alleviate the dependence on extensive manual annotations, FSL has emerged as an appealing paradigm for medical image analysis. Early FSL approaches, such as Prototypical Networks [37], learn a shared embedding space in which class prototypes derived from a small set of labeled support samples guide the prediction of query images. This idea has been widely adapted to medical image segmentation [38, 39], where prototype-based matching enables rapid adaptation to new tasks or domains with minimal supervision.

Building upon this paradigm, specialized architectures such as PANet [40] and ADNet [41] introduced prototype alignment and anomaly-aware mechanisms to improve the robustness of few-shot adaptation. Nevertheless, most existing medical FSL methods rely exclusively on visual similarity metrics defined over pixel intensities or low-level texture features [42, 43]. In challenging scenarios such as liver tumor segmentation from CT images—characterized by low contrast, heterogeneous appearance, and ambiguous boundaries—purely visual matching often fails to capture clinically meaningful distinctions. As a result, few-shot segmentation models frequently exhibit high sensitivity to noise and inter-case variability, limiting their reliability in real-world clinical settings.

Recent studies have also explored the adaptation of large-scale foundation models to few-shot medical tasks. For example, SAM [44] based variants such as SAMed [45] employ low-rank adaptation or prompt tuning to transfer general visual knowledge to medical domains. While these approaches benefit from strong generic representations, they largely operate within a visual-only framework and do not explicitly leverage the rich semantic information routinely used by clinicians during diagnosis and interpretation.

### 2.3 Vision–language models for clinical decision support

The emergence of large-scale VLMs has provided a new paradigm for integrating semantic knowledge into medical image analysis. By jointly learning visual and textual representations, models such as CLIP [46] and its medical extensions enable alignment between image features and high-level semantic concepts [47, 48], offering a form of representation that is more invariant to acquisition-specific variations. In digital health research, VLMs have been increasingly explored for tasks such as report generation [49], cross-modal retrieval [50], and clinical decision support [51], where language serves as a natural interface for encoding expert knowledge.

From a clinical perspective, language-based representations offer an interpretable and structured medium to capture diagnostic reasoning, including lesion morphology, spatial relationships, and contextual cues that extend beyond pixel-level appearance. However, existing VLM-based studies in medical imaging have primarily focused on classification or retrieval tasks, with limited investigation into their potential role in dense prediction problems such as segmentation—particularly under few-shot or low-annotation regimes.

This line of research suggests that incorporating language-derived semantic information may provide a complementary inductive bias for segmentation models, enabling more robust and clinically aligned representations. Yet, how to effectively integrate automated semantic guidance into few-shot medical image segmentation remains an open problem, motivating further exploration in this direction.

## 3 Materials and methods

### 3.1 Design rationale and framework overview

The proposed framework is designed to address two persistent challenges in digital hepatology: limited availability of annotated data and poor generalization across clinical centers due to domain shift [8, 36]. As discussed in the Introduction, conventional deep learning–based segmentation models predominantly rely on low-level pixel statistics, which are highly sensitive to variations in imaging protocols and scanner characteristics. In contrast, clinical interpretation by radiologists is guided by higher-level semantic understanding, which remains relatively invariant across acquisition conditions.

**Figure 1:**
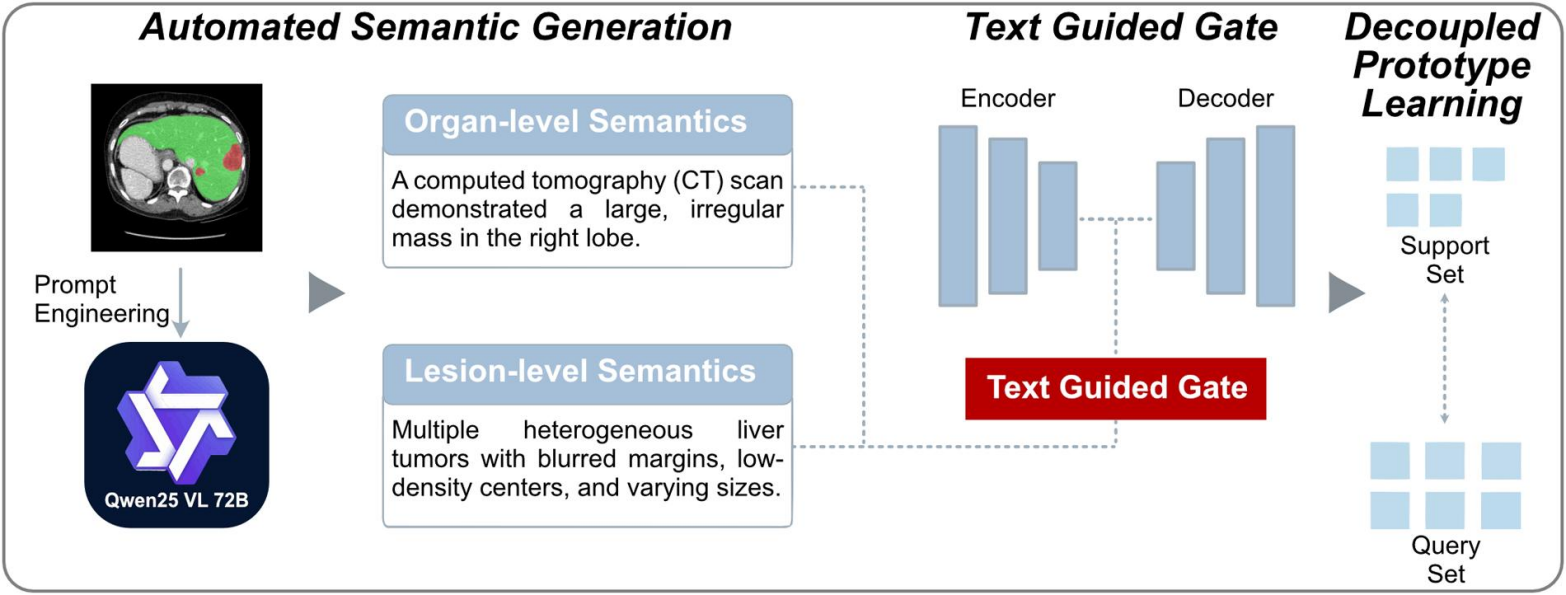
Overview of the proposed framework for semantic-guided few-shot liver tumor segmentation. The framework consists of three main components: (1) automated semantic generation using a vision–language model to extract organ- and lesion-level clinical descriptions; (2) semantic–visual feature fusion via a Text-Guided Gate (TGG); and (3) prototype learning for robust support–query matching in few-shot segmentation.

As shown in Figure 1, the proposed framework is designed to incorporate clinically meaningful semantic information as an explicit inductive bias for few-shot segmentation. Specifically, we hypothesize that language-derived semantic descriptors can serve as a stable “conceptual anchor,” enabling more robust feature representations under data scarcity and domain shift. This principle is operationalized through a motivation-driven framework consisting of three key components: (i) an **Automated Semantic Generator** to encode clinically structured semantic descriptions without manual annotation; (ii) a **Text-Guided Gate (TGG)** to modulate visual representations using semantic priors; and (iii) a **Decoupled Prototype Learner** to enable stable and unbiased few-shot adaptation.

The synergy between these modules follows a structured few-shot episodic training and inference paradigm. In each episode, the **Automated Semantic Generator** first extracts a hierarchical linguistic representation T from the annotated support images, acting as a global clinical prior for both the support and query branches. The **TGG** then functions as a multi-modal bridge, utilizing T to perform channel-wise modulation on the visual features extracted by the encoder, effectively filtering out domain-specific imaging noise. Finally, the **Decoupled Prototype Learner** extracts class-specific prototypes from the modulated support features through masked averaging and gradient detachment. These prototypes serve as stable reference anchors for similarity matching with the query features. During training, the entire framework is optimized end-to-end using a combined loss function that supervises both the coarse prototype matching and the refined decoder outputs, ensuring that the visual-semantic alignment is robust to the extreme class imbalances and domain shifts inherent in multi-center clinical data.

### 3.2 Automated semantic generation: encoding clinical expertise

To mitigate the limited availability of expert annotations in scalable clinical pipelines, we employ the Qwen2.5-VL-72B model as an automated pipeline for generating clinically structured semantic descriptions. Rather than performing generic image captioning, the semantic generator is guided to produce structured, hierarchical descriptions tailored to the challenges of liver tumor segmentation. This design ensures that the generated language representations align with clinically relevant reasoning processes.

Specifically, we extract two complementary levels of semantic information:

- **Organ-level semantics (liver-focused):** These descriptors capture global anatomical context, including liver shape, location, and spatial extent. Such information provides a robust localization prior that is less sensitive to domain-specific intensity variations and scanner-dependent artifacts [9].
- **Lesion-level semantics (tumor-focused):** Leveraging the available ground-truth masks in the support set, fine-grained descriptions are generated to characterize lesion appearance, boundary sharpness, and relative density patterns. These support-derived semantic cues then serve as a stable reference for the query images, explicitly designed to address boundary ambiguity and small target detection, which are common failure modes in few-shot liver tumor segmentation [2, 52].

By encoding clinical knowledge in a structured linguistic form, the automated semantic generator supplies concept-level priors that complement visual features and remain stable across heterogeneous datasets.

We evaluate two semantic granularity variants of the proposed framework: (i) **Ours (w/liver)**: the model is conditioned on organ-level semantic descriptions of the liver generated by the VLM; and (ii) **Ours (w/tumor)**: the model is conditioned on lesion-level semantic descriptions of the tumor. Both variants share identical base architecture and training configuration, differing solely in the granularity of the VLM-generated textual input.

To assess the semantic correctness of the generated descriptions, a random subset of 30 cases (15 LiTS, 15 3DIRCADb) was independently reviewed by two co-authors with clinical backgrounds in oncology and radiology, using a 3-point scale (correct/partially correct/incorrect). All organ-level descriptions (100%) and 93% of lesion-level descriptions were rated as fully correct; the remaining 7% were rated as partially correct. No descriptions were rated as clinically incorrect. These descriptions are intended to serve as approximate inductive priors that complement visual features, rather than as validated radiological reports suitable for direct clinical interpretation.

### 3.3 Text-guided gate

#### 3.3.1 CNN encoder with enhanced robustness

To extract discriminative visual representations while preserving fine-grained spatial details, we adopt a multi-stage convolutional encoder with L=5 hierarchical stages. Skip connections are employed to facilitate multi-scale feature aggregation, which is essential for accurate delineation of tumor boundaries. To improve robustness to inter-center variability and noise in CT imaging, Instance Normalization and LeakyReLU activations are used in place of Batch Normalization, as they have demonstrated superior stability in medical imaging scenarios with limited batch diversity.

#### 3.3.2 Text-guided gate for semantic modulation

To explicitly integrate semantic priors into the visual feature space, we introduce a TGG at the bottleneck layer of the network. Unlike conventional attention mechanisms, the TGG performs semantic-conditioned feature modulation, ensuring that the latent representation is actively shaped by language-derived clinical knowledge.

Let $f_{L}$ denote the visual feature map at the deepest encoder level and $z_{T}\in R^{D}$ represent the global text embedding. The gated feature representation is formally defined in Equation (1):

$$f_{L}^{\prime }=f_{L}⊙\sigma (W_{t}z_{T}+b_{t}),$$ (1)

where σ(⋅) denotes the sigmoid function. This gating operation acts as a semantic filter, suppressing background-dominated activations while amplifying features that align with the clinical description of liver anatomy and tumor characteristics.

### 3.4 Decoupled prototype learning

#### 3.4.1 Per-image prototype averaging for class imbalance

Liver tumor segmentation is inherently affected by severe class imbalance, as tumor regions may occupy less than 1% of an axial slice. Standard prototype learning approaches, which aggregate embeddings across all pixels, are therefore prone to size bias, where large background or organ regions dominate the prototype representation.

**Figure 2:**
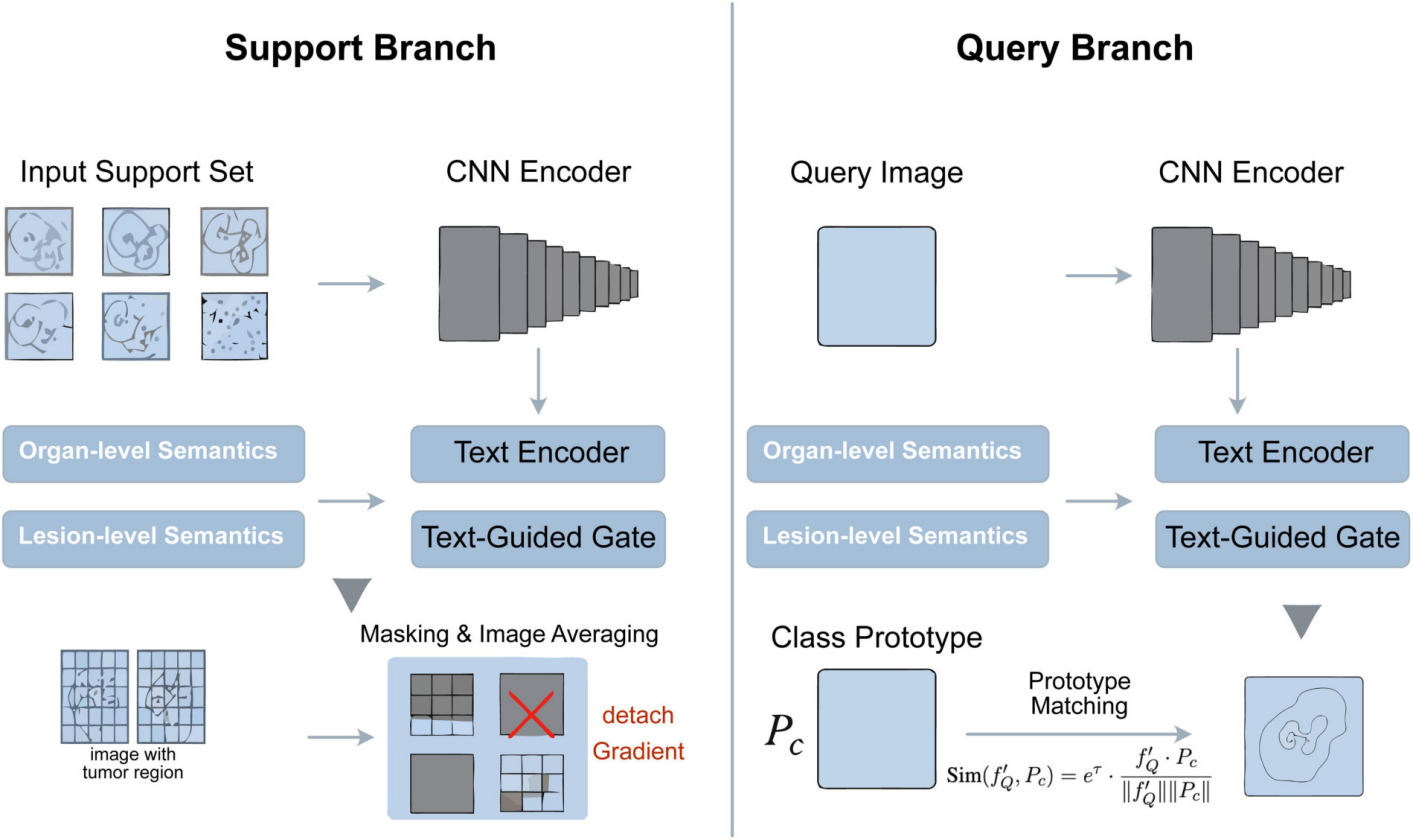
Support–query architecture with text-guided prototype learning for few-shot liver tumor segmentation. The support branch computes class prototypes through text-guided feature modulation and masked image averaging with gradient detachment, while the query branch performs similarity matching between query features and detached prototypes to generate the final segmentation mask.

As depicted in Figure 2, we adopt a per-image prototype averaging strategy. For each class c, the the prototype is formally given by Equation (2):

$$P_{c}=\frac{1}{N}\sum_{i=1}^{N}\text{Mean}(\{f_{L,i}^{\prime }(h,w)|(h,w)\in \Omega_{i,c}\}),$$ (2)

where $\Omega_{i,c}$ denotes the set of pixels belonging to class c in the i-th support image. By treating each support image as an independent semantic instance rather than a collection of pixels, this formulation ensures that small but clinically critical tumor regions contribute equally to the learned prototype.

#### 3.4.2 Gradient decoupling and CLIP-style similarity matching

To preserve the integrity of the few-shot learning setting and prevent information leakage between support and query branches, gradients are explicitly blocked from propagating through the prototypes, i.e., $P_{c}=\text{detach}(P_{c})$. This decoupling enforces a strict separation between prototype estimation and query adaptation.

Furthermore, inspired by CLIP-style contrastive learning, we employ a learnable logit scaling parameter τ to enhance discrimination in high-dimensional embedding spaces as expressed in Equation (3):

$$\text{Sim}(f_{Q}^{\prime }(h,w),P_{c})=e^{\tau }\cdot \frac{\;f_{Q}^{\prime }(h,w)\cdot P_{c}}{\left\|f_{Q}^{\prime }(h,w)\|\|P_{c}\right\|},$$ (3)

where (h,w) denotes the spatial coordinates of the feature map. This pixel-wise similarity formulation mitigates similarity saturation and improves class separability, particularly when distinguishing ambiguous tumor regions from surrounding liver tissue in external or cross-dataset evaluations.

## 4 Experiments

### 4.1 Datasets and preprocessing

We evaluate the proposed method on two publicly available liver CT datasets: LiTS and 3DIRCADb. LiTS is used as the primary dataset for episodic training and internal evaluation, while 3DIRCADb is used exclusively for cross-dataset external validation without any fine-tuning.

All volumes are used at their native acquisition spacing without resampling to a fixed resolution, and intensity-normalized using z-score normalization per volume. To ensure fair comparison across methods, identical preprocessing pipelines are applied to all models.

### 4.2 Few-shot segmentation protocol

We adopt a 1-way K-shot episodic segmentation setting. Unless otherwise specified, K=5 for all few-shot experiments.

During each evaluation episode:

- K support slices containing tumor regions are randomly sampled.
- One query slice is sampled from a different patient within the same dataset.
- Support and query images are processed independently, and similarity matching is performed via prototype comparison without any parameter fine-tuning.

For internal evaluation on LiTS, models are meta-trained on the training split, and evaluation episodes are sampled exclusively from the unseen test split at the patient level to avoid data leakage.

For external evaluation on 3DIRCADb, the models rely solely on the weights learned from the LiTS dataset without any further domain-specific retraining. Crucially, during this phase, both the K support samples and the query images are drawn exclusively from 3DIRCADb. This setup rigorously evaluates cross-dataset generalization and adaptation capability, measuring how effectively the model can segment unseen query cases in a new clinical domain guided by only K target-domain annotations.

### 4.3 Baseline configuration and fairness considerations

We compare our method against three categories of baselines:

1. **Fully Supervised Models.** UNet, nnU-Net, Attention U-Net, TransUNet, SwinUNETR, MedNeXt, and SegResNet are trained using full supervision on the complete LiTS training split. These models are evaluated directly on 3DIRCADb without domain adaptation to assess their zero-shot cross-dataset robustness.
2. **Foundation Model Variants.** SAMed and SAMed_light are fine-tuned on LiTS using full supervision following their official configuration.
3. **Few-Shot Segmentation Methods.** Vanilla_proto, ADNet, and PANet are trained and evaluated under the same episodic few-shot protocol as our method.

Importantly, during the external evaluation and deployment phase, few-shot methods (including ours) only require access to K annotated support samples from the target domain. In contrast, conventional supervised baselines either suffer from severe domain shift degradation or require extensive retraining on a fully annotated target dataset. This comparison fairly highlights the practical data efficiency and rapid adaptation capability of the proposed semantic-guided framework in real-world, multi-center clinical settings.

### 4.4 Implementation details

**Text encoder.** BioClinicalBERT is used frozen throughout training. The embedding is projected to 512 dimensions via a single trainable linear layer and fed into the TGG module.

**Visual encoder.** A 5-stage convolutional encoder with filter counts 32–64–128–256–512, 3 × 3 kernels, Instance Normalization, and LeakyReLU activations. Skip connections link each encoder stage to the corresponding decoder stage.

**TGG module.** A linear projection maps the 512-dim text embedding to a channel-wise sigmoid gating vector applied to the bottleneck feature map.

**Training.** AdamW optimizer, learning rate $1\times 10^{-4}$, weight decay $1\times 10^{-4}$, batch size 16,200 epochs, mixed-precision training, combined cross-entropy and Dice loss (equal weighting). Total trainable parameters: ≈15.27M (BioClinicalBERT frozen). Training is performed on a single NVIDIA RTX 3090Ti (24 GB VRAM), with peak memory usage of ≈4.6 GB.

**Semantic generation (Qwen2.5-VL-72B)** All textual descriptions are generated offline prior to training using Qwen2.5-VL-72B with deterministic greedy decoding (temperature =0.0, top-p=1.0, maximum 256 output tokens), and cached as JSON files to avoid any inference overhead during training. Two prompt templates are used depending on the semantic granularity level:

- **Organ-level prompt:** You are an expert radiologist analyzing a CT scan. Examine the liver region in this image and describe: (1) the overall shape and size of the liver; (2) the liver margin and boundary characteristics; (3) the liver parenchyma homogeneity; (4) the spatial position relative to adjacent structures. Provide a concise structured description in 2-3 sentences.
- **Lesion-level prompt:** You are an expert radiologist. A liver tumor has been annotated in this CT image (indicated by the overlay). Describe: (1) the tumor’s shape, size, and number; (2) its boundary sharpness and margin characteristics; (3) the attenuation/density pattern of the lesion relative to surrounding liver parenchyma; (4) any internal heterogeneity. Provide a concise, structured description in 2-3 sentences.

### 4.5 Quantitative results

Table 1 summarizes segmentation performance on internal (LiTS) and external (3DIRCADb) datasets. Table 2 further reports Precision, Recall, Specificity, and F1 scores across all methods and datasets.

**Table 1:** Quantitative comparison of segmentation performance (Dice, IoU, HD95) on LiTS (Internal) and 3DIRCADb (External). Results are reported as mean ± std, where standard deviations are shown in smaller font. HD95 is reported in millimeters (mm). Statistical significance is computed against Ours (w/tumor).

	Internal (LiTS)						External (3DIRCADb)						
	Liver			Tumor			Liver			Tumor			
Model	Dice	IoU	HD95 (mm)	Dice	IoU	HD95 (mm)	Dice	IoU	HD95 (mm)	Dice	IoU	HD95 (mm)	*p*-value
UNet	0.9758 ± 0.0147	0.9531 ± 0.0273	6.7991 ± 11.6635	0.8273 ± 0.2715	0.7673 ± 0.2714	10.7649 ± 23.8180	0.7427 ± 0.1923	0.6161 ± 0.1671	95.8846 ± 32.4249	0.2756 ± 0.2889	0.1973 ± 0.2248	80.9358 ± 48.5942	<0.05
nnU-Net	**0.9782** ± 0.0132	**0.9576** ± 0.0245	4.5025 ± 7.2098	0.8296 ± 0.2792	0.7735 ± 0.2747	7.4487 ± 16.1715	0.7514 ± 0.2101	0.6335 ± 0.1913	86.0234 ± 26.3930	0.2007 ± 0.2160	0.1293 ± 0.1486	70.5405 ± 41.7546	<0.05
Att-UNet	0.9759 ± 0.0152	0.9534 ± 0.0279	5.9048 ± 9.0327	0.8239 ± 0.2809	0.7660 ± 0.2780	11.4825 ± 23.8246	0.7288 ± 0.1897	0.5978 ± 0.1651	94.0120 ± 30.8357	0.2744 ± 0.3130	0.2037 ± 0.2474	80.7543 ± 47.4260	<0.05
TransUNet	0.9712 ± 0.0169	0.9445 ± 0.0311	11.3317 ± 13.7897	0.7730 ± 0.3092	0.7051 ± 0.3020	18.8938 ± 31.5857	0.7278 ± 0.1902	0.5970 ± 0.1687	91.9352 ± 31.3611	0.2857 ± 0.3160	0.2129 ± 0.2520	90.7933 ± 52.3378	<0.05
SwinUNETR	0.9749 ± 0.0130	0.9513 ± 0.0240	5.7797 ± 10.6562	0.8030 ± 0.2935	0.7417 ± 0.2941	12.7692 ± 22.7993	0.6805 ± 0.1864	0.5385 ± 0.1631	91.9096 ± 30.3845	0.1411 ± 0.1956	0.0898 ± 0.1328	115.2909 ± 44.4739	<0.05
MedNeXt	0.9776 ± 0.0129	0.9564 ± 0.0239	**3.9147** ± 3.0858	0.8387 ± 0.2711	**0.7846** ± 0.2711	**4.6692** ± 10.9545	0.5118 ± 0.2341	0.3748 ± 0.1983	90.2196 ± 31.4319	0.0677 ± 0.1311	0.0406 ± 0.0815	80.4438 ± 37.6291	<0.05
SegResNet	0.9752 ± 0.0157	0.9521 ± 0.0289	5.9505 ± 9.2055	0.8163 ± 0.2906	0.7588 ± 0.2852	8.8889 ± 18.1352	0.7706 ± 0.2067	0.6577 ± 0.1855	90.3345 ± 30.6018	0.2259 ± 0.2598	0.1558 ± 0.1962	64.8025 ± 34.6670	<0.05
SAMed	0.9329 ± 0.0278	0.8755 ± 0.0477	54.7376 ± 22.7401	0.7134 ± 0.2981	0.6206 ± 0.2909	40.6694 ± 39.9886	0.5288 ± 0.2445	0.3936 ± 0.2071	100.5049 ± 33.0574	0.0862 ± 0.1600	0.0536 ± 0.1032	125.6003 ± 37.3630	<0.05
SAMed-light	0.9333 ± 0.0278	0.8762 ± 0.0477	55.9740 ± 22.9976	0.7513 ± 0.2794	0.6629 ± 0.2794	37.7437 ± 44.2942	0.5669 ± 0.2170	0.4226 ± 0.1798	95.7725 ± 34.0098	0.0659 ± 0.1276	0.0393 ± 0.0794	120.2467 ± 35.6379	<0.05
UMamba	0.9718 ± 0.0155	0.9456 ± 0.0283	9.8697 ± 12.8541	0.7659 ± 0.3183	0.6997 ± 0.3106	15.2472 ± 24.2599	0.6977 ± 0.1851	0.5586 ± 0.1622	88.2806 ± 28.7623	0.2107 ± 0.2631	0.1466 ± 0.1968	80.0564 ± 31.0253	<0.05
Vanilla-Proto	0.9598 ± 0.0220	0.9235 ± 0.0391	28.9971 ± 34.7283	0.5576 ± 0.3725	0.4747 ± 0.3456	40.5917 ± 45.1185	0.7450 ± 0.1913	0.6192 ± 0.1688	90.3147 ± 31.4554	0.1407 ± 0.1706	0.0858 ± 0.1108	128.9627 ± 30.5211	<0.05
ADNet	0.9632 ± 0.0202	0.9297 ± 0.0365	16.0528 ± 23.0127	0.5954 ± 0.3701	0.5147 ± 0.3493	32.3793 ± 39.8688	0.4937 ± 0.1408	0.3374 ± 0.1042	115.0969 ± 32.5199	0.0675 ± 0.0826	0.0369 ± 0.0467	160.9990 ± 41.5655	<0.05
PANet	0.9657 ± 0.0188	0.9343 ± 0.0341	14.7835 ± 23.4711	0.5809 ± 0.3845	0.5058 ± 0.3602	30.1336 ± 40.2811	0.7224 ± 0.1971	0.5924 ± 0.1773	**73.7753** ± 33.7132	0.1660 ± 0.2813	0.1292 ± 0.2579	64.5411 ± 37.8964	<0.05
**Ours (w/ liver)**	0.9713 ± 0.0165	0.9446 ± 0.0304	9.9043 ± 17.3948	0.7731 ± 0.3080	0.7048 ± 0.3022	19.5821 ± 37.4303	**0.8161** ± 0.2103	**0.7249** ± 0.2026	80.9259 ± 37.8161	0.2714 ± 0.2569	0.1859 ± 0.1958	76.3137 ± 42.8599	–
**Ours (w/ tumor)**	0.9758 ± 0.0175	0.9533 ± 0.0321	9.8810 ± 20.3419	**0.8430** ± 0.2341	0.7770 ± 0.2447	11.4238 ± 26.6554	0.7837 ± 0.2026	0.6754 ± 0.1890	85.5361 ± 34.3185	**0.4056** ± 0.3265	**0.3138** ± 0.2948	**59.8927** ± 45.9279	–

**Bold** indicates the best value per column across all methods. We evaluate two semantic granularity variants of the proposed framework: (i) Ours (w/liver): the model is conditioned on organ-level semantic descriptions of the liver generated by the VLM; and (ii) Ours (w/tumor): the model is conditioned on lesion-level semantic descriptions of the tumor. Both variants share identical base architecture and training configuration, differing solely in the granularity of the VLM-generated textual input.

**Table 2:** Comparison of additional metrics (Precision, Recall, Specificity, F1) on LiTS (Internal) and 3DIRCADb (External) datasets. Results are reported as mean ± std, with reduced font size for standard deviation.

	Internal (LiTS)								External (3DIRCADb)							
	Liver				Tumor				Liver				Tumor			
Model	Prec	Rec	Spec	F1	Prec	Rec	Spec	F1	Prec	Rec	Spec	F1	Prec	Rec	Spec	F1
UNet	0.9610 ± 0.0236	0.9914 ± 0.0127	0.9926 ± 0.0044	0.9758 ± 0.0147	**0.7296** ± 0.3721	0.7064 ± 0.3747	0.9989 ± 0.0017	0.7119 ± 0.3686	0.6407 ± 0.1751	0.9474 ± 0.0335	0.9071 ± 0.0311	0.7427 ± 0.1923	0.2925 ± 0.3528	0.3393 ± 0.3041	0.9905 ± 0.0087	0.2756 ± 0.2889
nnU-Net	0.9650 ± 0.0189	0.9919 ± 0.0125	0.9934 ± 0.0034	**0.9782** ± 0.0132	0.7086 ± 0.3836	0.7040 ± 0.3821	0.9991 ± 0.0014	0.7046 ± 0.3804	0.7165 ± 0.2043	0.7938 ± 0.2205	0.9489 ± 0.0185	0.7514 ± 0.2101	0.3598 ± 0.3934	0.1871 ± 0.1904	0.9945 ± 0.0061	0.2007 ± 0.2160
Att-UNet	0.9613 ± 0.0234	0.9913 ± 0.0126	0.9927 ± 0.0039	0.9759 ± 0.0152	0.7191 ± 0.3768	0.7054 ± 0.3799	0.9990 ± 0.0015	0.7085 ± 0.3746	0.6246 ± 0.1741	0.9433 ± 0.0397	0.9010 ± 0.0330	0.7288 ± 0.1897	0.2951 ± 0.3665	0.3102 ± 0.3118	0.9907 ± 0.0083	0.2744 ± 0.3130
TransUNet	0.9543 ± 0.0266	0.9892 ± 0.0167	0.9912 ± 0.0052	0.9712 ± 0.0169	0.6772 ± 0.3801	0.6710 ± 0.3869	0.9984 ± 0.0025	0.6673 ± 0.3770	0.6341 ± 0.1792	0.9191 ± 0.0534	0.9095 ± 0.0293	0.7278 ± 0.1902	0.3171 ± 0.3726	0.3074 ± 0.3065	0.9908 ± 0.0147	0.2857 ± 0.3160
SwinUNETR	0.9605 ± 0.0181	0.9900 ± 0.0164	0.9925 ± 0.0034	0.9749 ± 0.0130	0.6818 ± 0.3945	0.6668 ± 0.3913	0.9991 ± 0.0015	0.6683 ± 0.3868	0.5972 ± 0.1740	0.8306 ± 0.1308	0.9018 ± 0.0339	0.6805 ± 0.1864	0.1644 ± 0.2666	0.1869 ± 0.2435	0.9811 ± 0.0085	0.1411 ± 0.1956
MedNeXt	0.9637 ± 0.0197	0.9921 ± 0.0106	0.9932 ± 0.0033	0.9776 ± 0.0129	0.7167 ± 0.3791	0.7165 ± 0.3819	**0.9992** ± 0.0011	**0.7137** ± 0.3775	0.5466 ± 0.2054	0.5169 ± 0.2792	0.9246 ± 0.0345	0.5118 ± 0.2341	0.1758 ± 0.3016	0.0453 ± 0.0933	0.9957 ± 0.0050	0.0677 ± 0.1311
SegResNet	0.9603 ± 0.0247	0.9910 ± 0.0134	0.9926 ± 0.0039	0.9752 ± 0.0157	0.6952 ± 0.3787	0.7131 ± 0.3884	0.9989 ± 0.0016	0.7009 ± 0.3796	0.6966 ± 0.1962	0.8860 ± 0.1650	0.9343 ± 0.0212	0.7706 ± 0.2067	0.2579 ± 0.3239	0.2321 ± 0.2416	0.9814 ± 0.0182	0.2259 ± 0.2598
SAMed	0.8859 ± 0.0478	0.9870 ± 0.0171	0.9746 ± 0.0159	0.9329 ± 0.0278	0.6109 ± 0.3427	0.6248 ± 0.3833	0.9957 ± 0.0086	0.5980 ± 0.3532	0.4880 ± 0.1870	0.6106 ± 0.3269	0.8916 ± 0.0460	0.5288 ± 0.2445	0.0767 ± 0.1487	0.1270 ± 0.1870	0.9537 ± 0.0322	0.0862 ± 0.1600
SAMed-light	0.8859 ± 0.0474	0.9878 ± 0.0168	0.9747 ± 0.0159	0.9333 ± 0.0278	0.6331 ± 0.3576	0.6256 ± 0.3794	0.9963 ± 0.0081	0.6167 ± 0.3572	0.5153 ± 0.1742	0.6589 ± 0.2945	0.8891 ± 0.0447	0.5669 ± 0.2170	0.0705 ± 0.1307	0.1329 ± 0.1800	0.9680 ± 0.0275	0.0659 ± 0.1276
UMamba	0.9547 ± 0.0245	0.9900 ± 0.0155	0.9913 ± 0.0043	0.9718 ± 0.0155	0.6863 ± 0.3837	0.6672 ± 0.3862	0.9988 ± 0.0017	0.6698 ± 0.3784	0.6405 ± 0.1803	0.8300 ± 0.0941	0.9205 ± 0.0275	0.6977 ± 0.1851	0.2761 ± 0.3709	0.2754 ± 0.2799	0.9865 ± 0.0108	0.2107 ± 0.2631
Vanilla-Proto	0.9466 ± 0.0353	0.9745 ± 0.0256	0.9893 ± 0.0082	0.9598 ± 0.0220	0.5220 ± 0.3893	0.5330 ± 0.3943	0.9966 ± 0.0051	0.5095 ± 0.3768	0.6361 ± 0.1764	0.9668 ± 0.0276	0.9035 ± 0.0354	0.7450 ± 0.1913	0.0882 ± 0.1156	0.6942 ± 0.4144	0.8589 ± 0.0475	0.1407 ± 0.1706
ADNet	0.9537 ± 0.0285	0.9739 ± 0.0300	0.9911 ± 0.0060	0.9632 ± 0.0202	0.5482 ± 0.3903	0.5435 ± 0.3967	0.9969 ± 0.0050	0.5281 ± 0.3811	0.3374 ± 0.1042	**1.0000** ± 0.0001	0.6372 ± 0.0577	0.4937 ± 0.1408	0.0371 ± 0.0469	**0.7458** ± 0.3828	0.6497 ± 0.0904	0.0675 ± 0.0826
PANet	0.9573 ± 0.0258	0.9752 ± 0.0285	0.9917 ± 0.0054	0.9657 ± 0.0188	0.5796 ± 0.4065	0.5287 ± 0.3968	0.9977 ± 0.0034	0.5232 ± 0.3922	**0.7716** ± 0.2140	0.7095 ± 0.1486	**0.9657** ± 0.0180	0.7224 ± 0.1971	0.3205 ± 0.4431	0.0662 ± 0.1170	**0.9994** ± 0.0011	0.0993 ± 0.1736
**Ours (w/liver)**	0.9539 ± 0.0255	0.9898 ± 0.0178	0.9912 ± 0.0049	0.9713 ± 0.0165	0.6750 ± 0.3914	0.6522 ± 0.3826	0.9989 ± 0.0017	0.6577 ± 0.3802	0.7489 ± 0.2151	0.9550 ± 0.0451	0.9453 ± 0.0323	**0.8161** ± 0.2103	0.3077 ± 0.3484	0.3382 ± 0.2903	0.9896 ± 0.0105	0.2714 ± 0.2569
**Ours (w/tumor)**	**0.9665** ± 0.0263	0.9858 ± 0.0196	**0.9937** ± 0.0050	0.9758 ± 0.0175	0.7191 ± 0.3727	0.6910 ± 0.3687	0.9991 ± 0.0014	0.6988 ± 0.3646	0.7147 ± 0.2011	0.9358 ± 0.0453	0.9388 ± 0.0260	0.7837 ± 0.2026	**0.3468** ± 0.3437	0.4325 ± 0.3393	0.9877 ± 0.0118	**0.3390** ± 0.2993

**Bold** indicates the best value per column across all methods. We evaluate two semantic granularity variants of the proposed framework: (i) Ours (w/liver): the model is conditioned on organ-level semantic descriptions of the liver generated by the VLM; and (ii) Ours (w/tumor): the model is conditioned on lesion-level semantic descriptions of the tumor. Both variants share identical base architecture and training configuration, differing solely in the granularity of the VLM-generated textual input.

On the internal dataset, fully supervised models achieve strong liver segmentation performance (Dice > 0.97), while tumor segmentation remains more challenging. Our model with tumor-level semantic guidance achieves the highest tumor Dice (0.8430) under the few-shot setting.

On the external dataset, most supervised models exhibit substantial performance degradation, particularly for tumor segmentation. For example, MedNeXt shows a drop from 0.8387 to 0.0677 in tumor Dice, indicating sensitivity to domain shift.

In contrast, our method demonstrates improved cross-dataset robustness. Under tumor-level semantic guidance, the proposed framework achieves a tumor Dice of 0.4015 on 3DIRCADb, outperforming all few-shot baselines and several fully supervised models. Compared to PANet (0.1660), this corresponds to approximately a 2.6× relative improvement.

Notably, HD95 for tumor segmentation is reduced to 59.89 mm on the external dataset, indicating improved boundary stability under domain shift.

### 4.6 Ablation study: impact of semantic granularity

To evaluate the contribution of semantic granularity, Table 3 compares incremental configurations from the vanilla prototype baseline to the full model.

**Table 3:** Ablation study on 5-shot setting (mean ± std over 5 seeds).

Configuration	Internal (LiTS)		External (3DIRCADb)	
	Liver dice	Tumor dice	Liver dice	Tumor dice
Vanilla Prototype (baseline)	0.9598±0.0220	0.5576±0.3725	0.7450±0.1913	0.1407±0.1706
+ Per-Image Averaging	0.9611±0.0102	0.5924±0.0114	0.7251±0.0089	0.1214±0.0106
+ Gradient Detachment	0.9690±0.0091	0.6345±0.0108	0.7624±0.0112	0.2150±0.0085
+ TGG (Liver-level)	0.9713±0.0165	0.7731±0.3080	0.8161±0.2103	0.2714±0.2569
+ TGG (Tumor-level)	0.9758±0.0175	0.8430±0.2341	0.7837±0.2026	0.4056±0.3265

While liver-level semantics improve global localization robustness (external liver Dice = 0.8152), tumor-level semantics provide stronger gains in tumor segmentation performance (Dice = 0.4015). This suggests that fine-grained lesion descriptions serve as more effective inductive priors for small-target adaptation.

These findings support the hypothesis that language-derived semantic information can complement visual similarity metrics in few-shot medical image segmentation. Table 4 reports K-shot sensitivity analysis on both datasets, showing that performance generally improves from K=1 to K=3 before plateauing, demonstrating robustness across different support set sizes.

**Table 4:** K-shot sensitivity analysis on external (3DIRCADb) dataset (mean ± std over 5 seeds).

K	Internal (LiTS)		External (3DIRCADb)	
	Liver dice	Tumor dice	Liver dice	Tumor dice
1	0.9680±0.0009	0.6712±0.0478	0.7361±0.0117	0.2209±0.0216
2	0.9680±0.0029	0.6972±0.0078	0.7363±0.0147	0.2909±0.0336
3	0.9682±0.0020	0.7153±0.0046	0.7365±0.0406	0.3468±0.0625
5	0.9694±0.0008	0.7018±0.0447	0.7471±0.0308	0.3830±0.1479
10	0.9670±0.0016	0.6823±0.0222	0.7480±0.0018	0.2884±0.0641

### 4.7 Qualitative visualization

**Figure 3:**
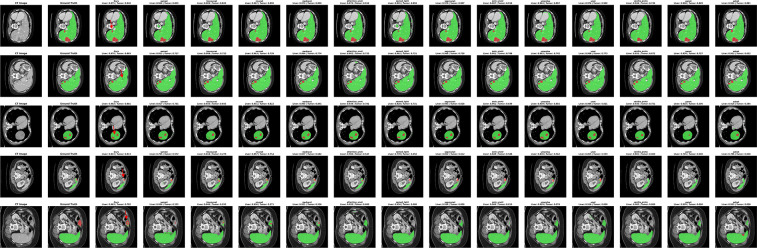
Visual comparison of representative segmentation methods on the internal dataset.

**Figure 4:**
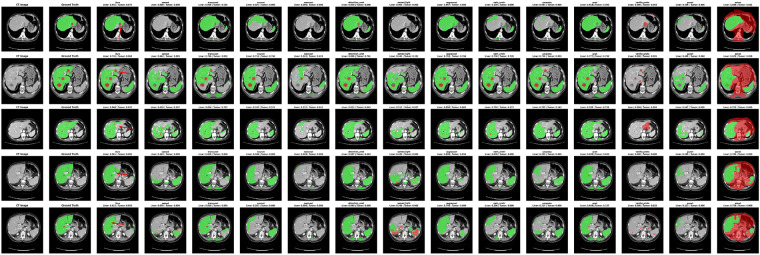
Visual comparison of representative segmentation methods on the external dataset.

Figures 3, 4 present representative qualitative visualization on the internal (LiTS) and external (3DIRCADb) datasets, respectively. On the internal cohort, most supervised models are able to delineate the overall liver structure; however, noticeable discrepancies remain in tumor boundary definition, particularly for small or irregular lesions. In contrast, our method produces more coherent and anatomically consistent segmentation masks, with improved delineation of complex tumor margins.

The performance gap becomes more pronounced under external validation. Several baseline models exhibit fragmented predictions, boundary leakage, or missed small lesions when confronted with domain shifts. Visual-only few-shot approaches, in particular, tend to generate unstable tumor contours and false positives in heterogeneous parenchymal regions. By incorporating semantic guidance, our framework maintains clearer boundary adherence and better captures subtle density variations, resulting in segmentation masks that align more closely with radiological interpretation. Overall, the qualitative comparisons corroborate the quantitative findings, demonstrating that text-guided semantic modulation enhances cross-dataset robustness and improves fine-grained pathological delineation under limited supervision.

## 5 Discussion

The integration of VLMs into medical image analysis pipelines reflects a broader paradigm shift from purely data-driven learning toward knowledge-informed clinical AI. In this study, we demonstrate that high-level semantic descriptors can serve as effective *conceptual anchors*, enabling robust few-shot liver tumor segmentation under severe data scarcity and cross-domain distribution shift. By explicitly bridging pixel-level visual representations with clinically structured semantic descriptors, the proposed framework offers a scalable solution for deploying segmentation models across heterogeneous clinical environments without relying on extensive manual re-annotation.

A key insight from our experiments is that the *granularity of semantic guidance* fundamentally determines the model’s generalization behavior. Organ-level descriptions primarily function as coarse spatial priors, stabilizing global localization across datasets. In contrast, lesion-level semantics that encode density characteristics and boundary clarity are critical for capturing pathologically meaningful details. This distinction is particularly relevant in digital oncology, where tumor appearance is highly sensitive to contrast phase, acquisition protocol, and scanner-specific artifacts. The stable external performance observed in our framework suggests that semantic priors are inherently less susceptible to low-level imaging noise than conventional supervised features, making them well suited for real-world clinical deployment where heterogeneity is unavoidable.

Beyond segmentation accuracy, our work highlights the practical significance of *automated semantic acquisition*. By leveraging Qwen2.5-VL to generate structured textual descriptions, the framework alleviates a major bottleneck in clinical AI workflows: the dependence on manual expert annotations for contextual knowledge. As an automated semantic extraction mechanism that reduces reliance on manual annotation, the VLM extracts clinically meaningful abstractions that guide downstream segmentation without increasing the annotation burden. This design naturally aligns with a future *human-in-the-loop* paradigm, where AI systems propose initial semantic hypotheses that can be selectively refined by clinicians, achieving an efficient balance between automation and expert oversight.

**Figure 5:**
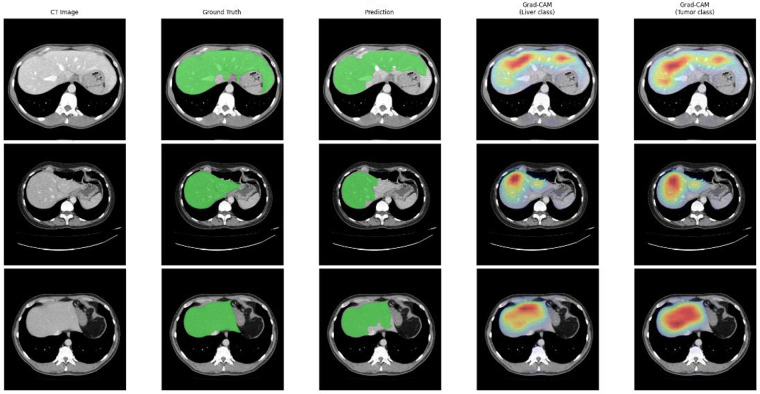
Grad-CAM activation maps for three representative cases. From left to right: CT image, ground truth mask, predicted segmentation, Grad-CAM (liver class), and Grad-CAM (tumor class). Liver-class activations (column 4) are broadly distributed yet anatomically confined to the hepatic region, while tumor-class activations (column 5) are more localized, with high-activation regions closely corresponding to tumor boundaries in the ground truth. The absence of diffuse or spurious activations in surrounding structures provides interpretable evidence that performance gains stem from semantically guided feature modulation rather than incidental correlations.

Despite its promising cross-center performance, several limitations remain. First, the framework relies on static textual descriptors and processes 2D axial slices independently, which may not fully capture three-dimensional spatial continuity or temporal tumor evolution. Second, the external validation set (3DIRCADb) comprises only 20 CT volumes, so cross-dataset results should be interpreted as indicative rather than definitive. Third, the current framework does not support Couinaud segment-level localization, intrahepatic/extrahepatic tumor distinction, or vascular involvement assessment, all of which require explicit 3D anatomical modeling beyond the present scope and are identified as directions for future work. Finally, extending the proposed approach to other low-contrast pathologies such as pancreatic or renal tumors will further test its generality and clinical value. As shown in Figure 5, Grad-CAM activation maps confirm that performance gains stem from semantically guided feature modulation rather than incidental correlations.

Overall, this study suggests that reframing medical image segmentation as a *knowledge-guided reasoning process*, rather than a purely visual matching task, can substantially improve robustness and data efficiency. Such a perspective is central to the long-term vision of digital health, where AI systems must generalize reliably across institutions, populations, and imaging protocols.

## 6 Conclusion

This work presents a text-guided few-shot segmentation framework that directly addresses two persistent challenges in digital hepatology: limited annotated data and pronounced domain shift across clinical centers. By augmenting visual feature learning with automated semantic descriptors generated by large vision–language models, we transform segmentation from a purely visual matching problem into a knowledge-guided reasoning process. The resulting semantic regularization provides a domain-invariant conceptual anchor that stabilizes performance in unseen clinical environments.

Our findings demonstrate that fine-grained pathological semantics are essential for accurate delineation of heterogeneous liver tumors, particularly under cross-dataset evaluation. The combination of decoupled prototype learning and CLIP-style similarity matching further contributes to training stability and practical robustness, supporting the feasibility of clinical deployment.

More broadly, this study illustrates the potential of integrating automated clinical knowledge into data-efficient medical imaging models. As digital health systems increasingly prioritize scalability and generalizability, the synergy between visual perception and language-based clinical abstraction offers a promising pathway toward improving data efficiency and generalizability in medical image segmentation. Future research will extend this multi-modal paradigm to additional disease sites and longitudinal clinical settings, further advancing the development of data-efficient and generalizable medical image segmentation models.

## Code availability

Upon acceptance, a complete implementation will be publicly released, including: (1) all model code (text-guided segmentation model, TGG module, decoupled prototype learner); (2) training, evaluation, and inference scripts; (3) prompt templates and all generated organ- and lesion-level textual descriptions for LiTS and 3DIRCADb; (4) exact case-level data splits with corresponding case IDs; and (5) pretrained model weights for all reported K-shot settings (K∈{1,3,5,10}). The repository link will be inserted upon acceptance. https://github.com/jcwang123/liver_seg.

## Data Availability

The original contributions presented in the study are included in the article/Supplementary Material, further inquiries can be directed to the corresponding author.
